# Statin Use and COVID-19 Infectivity and Severity in South Korea: Two Population-Based Nationwide Cohort Studies

**DOI:** 10.2196/29379

**Published:** 2021-10-08

**Authors:** Seung Won Lee, So Young Kim, Sung Yong Moon, In Kyung Yoo, Eun-Gyong Yoo, Gwang Hyeon Eom, Jae-Min Kim, Jae Il Shin, Myung Ho Jeong, Jee Myung Yang, Dong Keon Yon

**Affiliations:** 1 Department of Data Science Sejong University College of Software Convergence Seoul Republic of Korea; 2 Department of Otorhinolaryngology-Head & Neck Surgery CHA Bundang Medical Center CHA University School of Medicine Seongnam Republic of Korea; 3 Department of Gastroenterology CHA Bundang Medical Center CHA University School of Medicine Seongnam Republic of Korea; 4 Department of Pediatrics CHA Bundang Medical Center CHA University School of Medicine Seongnam Republic of Korea; 5 Department of Pharmacology Chonnam National University Medical School Hwasun Republic of Korea; 6 Department of Psychiatry Chonnam National University Hospital Chonnam National University Medical School Gwangju Republic of Korea; 7 Department of Pediatrics Severance Hospital Yonsei University College of Medicine Seoul Republic of Korea; 8 The Heart Center Chonnam National University Hospital Chonnam National University Medical School Gwangju Republic of Korea; 9 Department of Ophthalmology Asan Medical Center University of Ulsan College of Medicine Seoul Republic of Korea; 10 Department of Pediatrics Seoul National University Hospital Seoul National University College of Medicine Seoul Republic of Korea

**Keywords:** COVID-19, statin, susceptibility, severe clinical outcomes, length of hospital stay

## Abstract

**Background:**

Basic studies suggest that statins as add-on therapy may benefit patients with COVID-19; however, real-world evidence of such a beneficial association is lacking.

**Objective:**

We investigated differences in SARS-CoV-2 test positivity and clinical outcomes of COVID-19 (composite endpoint: admission to intensive care unit, invasive ventilation, or death) between statin users and nonusers.

**Methods:**

Two independent population-based cohorts were analyzed, and we investigated the differences in SARS-CoV-2 test positivity and severe clinical outcomes of COVID-19, such as admission to the intensive care unit, invasive ventilation, or death, between statin users and nonusers. One group comprised an unmatched cohort of 214,207 patients who underwent SARS-CoV-2 testing from the Global Research Collaboration Project (GRCP)-COVID cohort, and the other group comprised an unmatched cohort of 74,866 patients who underwent SARS-CoV-2 testing from the National Health Insurance Service (NHIS)-COVID cohort.

**Results:**

The GRCP-COVID cohort with propensity score matching had 29,701 statin users and 29,701 matched nonusers. The SARS-CoV-2 test positivity rate was not associated with statin use (statin users, 2.82% [837/29,701]; nonusers, 2.65% [787/29,701]; adjusted relative risk [aRR] 0.97; 95% CI 0.88-1.07). Among patients with confirmed COVID-19 in the GRCP-COVID cohort, 804 were statin users and 1573 were matched nonusers. Statin users were associated with a decreased likelihood of severe clinical outcomes (statin users, 3.98% [32/804]; nonusers, 5.40% [85/1573]; aRR 0.62; 95% CI 0.41-0.91) and length of hospital stay (statin users, 23.8 days; nonusers, 26.3 days; adjusted mean difference –2.87; 95% CI –5.68 to –0.93) than nonusers. The results of the NHIS-COVID cohort were similar to the primary results of the GRCP-COVID cohort.

**Conclusions:**

Our findings indicate that prior statin use is related to a decreased risk of worsening clinical outcomes of COVID-19 and length of hospital stay but not to that of SARS-CoV-2 infection.

## Introduction

COVID-19 is caused by SARS-CoV-2, and started in Wuhan, China. The World Health Organization (WHO) declared COVID-19 a pandemic on March 12, 2020 [1,2]. In Korea, the first COVID-19 patient was diagnosed on January 20, 2020. During the period from January 1, 2020, to May 31, 2020, the number of daily confirmed COVID-19 cases was less than 2000 and the cumulative number of COVID-19 cases was 11,468 with 270 deaths [3,4]. During this pandemic phase, efficient strategies for triage and therapeutics are crucial due to the high number of patients with SARS-CoV-2 infection, and the relatively limited facilities and medical resources [1,2]. The effective treatment of patients with COVID-19 has still not been established. Previous studies have suggested potential therapeutic candidates, including antimalarial drugs [5], antivirals such as lopinavir and ritonavir in combination [6], remdesivir [7], previous bacillus Calmette–Guérin (BCG) vaccination [8], famotidine [9], and immunoglobulin-containing sera from convalescent patients with COVID-19 [10]. However, antimalarial drugs and the lopinavir–ritonavir combination proved ineffective in clinical trials [5,6], and previous studies on BCG vaccination, famotidine, and immunoglobulin-containing sera had small sample sizes and preliminary study designs [8-10]. Remdesivir improved clinical outcomes in patients with COVID-19, but it is not readily available [7].

In this context, statins are inexpensive and easily available therapeutic agents, and their multiple pharmacologic mechanisms include anti-inflammation, antioxidation, inhibition of the angiotensin-converting enzyme 2 (ACE2) pathway, and lowering bodily lipid levels [11]. Statins are recommended for the primary prevention of cardiovascular diseases according to the American College of Cardiology/American Heart Association guidelines [12], and a recent retrospective cohort study has reported on their protective roles in preventing both all-cause mortality and cardiovascular-related mortality in the elderly [13]. The pleiotropic effects of statin on anti-inflammation and immune modulation, in addition to their inhibition of viral entry via ACE2, indicate the potential beneficial effects of statins on patients with COVID-19 [11,14]. In line with this, a recent study on patients with COVID-19 presented a negative association between statin use and risks of all-cause mortality [15]; however, the study was limited by the relatively small study population and the potential selection bias due to the unconcerned variables including lifestyle factors such as obesity, smoking, and alcohol consumption.

We hypothesized that prior statin use could either decrease the risks of COVID-19 or of severe clinical outcomes of COVID-19 (ie, death, admission to the intensive care unit, and invasive ventilation). Through 2 independent nationwide cohort studies on Korean patients, with propensity score matching, we investigated the potential association of previous statin use with the likelihood of a positive SARS-CoV-2 test result (viral infectivity) in all patients who underwent the test. Furthermore, we aimed to clarify the difference in clinical outcomes of patients with laboratory-confirmed SARS-CoV-2 infection who were and who were not administered statins.

## Methods

### Study Design

Two independent cohorts were analyzed: the Global Research Collaboration Project on COVID-19 (GRCP-COVID) cohort [16,17] and the National Health Insurance Service-COVID-19 (NHIS-COVID) cohort. The study protocol was approved by the Institutional Review Board of Sejong University (SJU-HR-E-2020-003). The requirement for written informed consent was waived by the ethics committee due to the urgent medical needs during the COVID-19 pandemic.

### GRCP-COVID Cohort (Claims-Based Cohort)

During the COVID-19 pandemic, the Korean Government shared the first nationwide claims-based database consisting of all people who were tested for SARS-CoV-2 in South Korea. This high-quality, large-scale nationwide cohort included all people who tested through medical or Korea Centers for Disease Control referrals (excluding self-referral) in South Korea via services facilitated by the Health Insurance Review and Assessment Service of Korea, the Korea Centers for Disease Control and Prevention, and the Ministry of Health and Welfare, Republic of Korea [16,18,19]. This cohort study has the following characteristics: (1) The Korean Government provided obligatory and complimentary medical health insurance for all patients with COVID-19; (2) therefore, this database has records of personal data, health care records of inpatients and outpatients for 3 years before the first SARS-CoV-2 test (including health care visits, prescriptions, diagnoses, and procedures), pharmaceutical visits, COVID-19-associated outcomes, and death records; and (3) all claim-based data were anonymous to maintain patient confidentiality with the Ministry of Health and Welfare and the Korean Government.

We identified all patients older than 20 years who underwent tests for SARS-CoV-2 infection in South Korea between January 1, 2020, and May 15, 2020 (n=214,207). As the pathophysiology of COVID-19 differs between children and adults, we excluded pediatric patients from the analysis [20]. The positive SARS-CoV-2 test results were based on real-time reverse transcriptase-polymerase chain reaction assays of nasal or pharyngeal swabs, following the WHO guidelines [8]. For each identified patient who was tested for SARS-CoV-2 infection, the cohort entry data (individual index data) included the date of the first SARS-CoV-2 test. Health care records of inpatients and outpatients between January 1, 2017, and May 15, 2020, were combined, and personal data on the age, sex, and region of residence were extracted from the insurance eligibility data.

A history of diabetes mellitus, cardiovascular disease, cerebrovascular disease, chronic obstructive pulmonary disease (COPD), hypertension, or chronic kidney disease was identified in at least two claims of inpatients or outpatients, or both, within 1 year using the appropriate International Classification of Disease 10th revision (ICD-10) codes [16,18]. The Charlson Comorbidity Index score was calculated using ICD-10 codes, as reported previously [21]. Use of medication (aspirin, metformin, and systemic glucocorticoids) was defined as taking any of these medications at 1-30 days before the index data. Demographics such as age, sex, and region of residence were obtained from the insurance eligibility data. The region of residence was classified as either urban (Seoul, Sejong, Busan, Incheon, Daegu, Gwangju, Daejeon, and Ulsan) or rural (Gyeonggi, Gangwon, Gyeongsangbuk, Gyeongsangnam, Chungcheongbuk, Chungcheongnam, Jeollabuk, Jeollanam, and Jeju) [22].

### NHIS-COVID-19 Cohort (Interview-Based Cohort)

Data were from individuals aged 20 years or older who underwent a SARS-CoV-2 test through a medical or Korea Centers for Disease Control referral (excluding self-referral) between January 1, 2020, and May 31, 2020, or through a general health examination between January 1, 2019, and December 31, 2019, as registered by the National Health Insurance Service of Korea (n=74,866).

Baseline information was obtained for each individual at the time of the general health examination. A history of diabetes mellitus, stroke, or cardiovascular disease; previous use of medication for hypertension, diabetes mellitus, or cardiovascular disease; smoking habit; physical activity; and frequency of alcohol consumption were obtained via self-reported questionnaires [21]. Body mass index and blood pressure were measured; data on serum glucose, creatinine, total cholesterol, low-density lipoprotein-cholesterol, and high-density lipoprotein-cholesterol were obtained from fasting blood samples [21].

### Exposure

We identified all lipophilic (atorvastatin, simvastatin, fluvastatin, lovastatin, and cerivastatin) and hydrophilic (rosuvastatin and pravastatin) statins, prescribed within 1 year before the index data [23]. Statin users were defined as patients who took statins 1-30 days before the index data. Patients who received statins 31-365 days before the index data were excluded [24]. Nonusers were defined as patients who did not take statins 1-365 days before the index data.

### Outcomes

The primary outcome was a laboratory-confirmed SARS-CoV-2 positivity, among all patients who were tested. The secondary outcomes were severe outcomes of COVID-19 [25], consisting of intensive care unit admission, invasive ventilation, or death, among patients who tested positive for SARS-CoV-2.

### Statistical Analysis

In the GRCP-COVID cohort, we performed each propensity score matching twice to compare SARS-CoV-2 test positivity (primary outcome) with severe clinical outcomes of patients with COVID-19 (secondary outcome), to minimize potential confounding factors and balance the baseline covariates of the 2 groups. First, we assessed the predicted probability of statin users versus nonusers among patients who underwent the SARS-CoV-2 test (n=214,207) using a logistic regression model with adjustment for potential confounding factors by age; sex; region of residence (rural or urban); a history of diabetes mellitus, cardiovascular disease, cerebrovascular disease, COPD, hypertension, or chronic kidney disease; Charlson Comorbidity Index (0, 1, or ≥2); use of aspirin, metformin, or systemic glucocorticoids. Second, we assessed the predicted probability of statin users versus nonusers among patients who tested positive for SARS-CoV-2 (n=7566) with the aforementioned adjustments for potential confounding factors. We performed the matching in the 2 groups in a 1:1 or 1:2 ratio using a “greedy nearest-neighbor” algorithm among all individuals who underwent the SARS-CoV-2 test and among those who tested positive for SARS-CoV-2, respectively, using random selection without replacement within caliper widths of 0.01 SDs.

In the NHIS-COVID cohort, we performed each propensity score matching twice, using the same methods as those used for the GRCP-COVID cohort, with adjustment for potential confounding factors by age (20-59, 60-69, and ≥70 years); sex; region of residence; a history of diabetes mellitus, stroke, cardiovascular disease; Charlson Comorbidity Index; body mass index (<25, 25-30, and ≥30 kg/m^2^); systolic blood pressure (continuous); diastolic blood pressure (continuous); fasting blood glucose (continuous); serum total cholesterol (continuous); serum low-density lipoprotein-cholesterol (continuous); serum high-density lipoprotein-cholesterol (continuous); estimated glomerular filtration rate (normal, mildly decreased, and moderately to severely decreased); household income (low, middle, and high); smoking status (never smoker, ex-smoker, and current smoker); frequency of alcohol consumption (<1, 1-2, 3-4, 5-6, and 7 times per week); physical activity (0, 1-2, 3-4, 5-6, and 7 sessions per week); and medication for hypertension, diabetes mellitus, or cardiovascular disease.

Adequate propensity score matching was confirmed by comparing propensity score densities (Multimedia Appendix 1) and standardized mean differences (SMDs) [23]. This approach assessed by SMDs is more meaningful than assessing *P* values from *t* tests [21,23]. Statistical analyses were performed using SAS version 9.4 (SAS Institute Inc.) and R software version 3.1.1 (R Foundation). Two-sided *P* values <.05 were considered statistically significant.

### Main Analysis

The “exposure” considered the current use of statin, and the “primary endpoint” was the positive test results for SARS-CoV-2 among all patients who were tested for SARS-CoV-2. The “secondary endpoint” was the severe clinical outcomes and the length of hospital stay among patients with laboratory-confirmed COVID-19. Data were analyzed using modified Poisson regression models, and adjusted relative risks (aRRs) with 95% CIs for the 2 groups in each propensity score–matched cohort were estimated after adjusting for potential covariates.

## Results

### Unmatched GRCP-COVID Cohort

**Figure 1:**
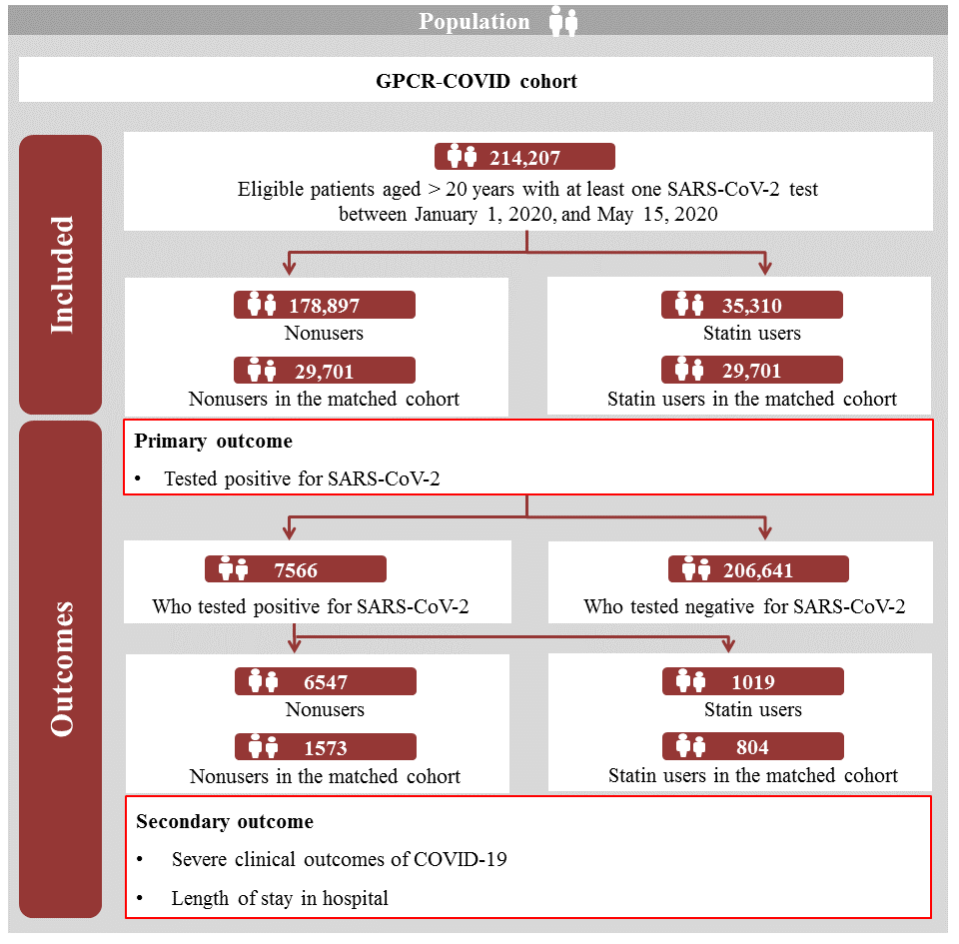
Graphical depiction of patient enrollment in the GRCP-COVID cohort (South Korea; January 1 to May 15, 2020). GRCP: Global Research Collaboration Project.

Among patients who underwent SARS-CoV-2 testing (n=214,207), we identified 178,897 nonusers and 35,310 statin users in the full unmatched cohort (Table 1 and Figure 1 and Multimedia Appendix 1). Among patients with laboratory-confirmed COVID-19 (n=7566), we identified 6547 nonusers and 1019 statin users in the full unmatched cohort.

**Table 1:** Baseline characteristics of all patients who were tested for SARS-CoV-2 infection and of those with laboratory-confirmed SARS-CoV-2 infection in the GRCP^a^-COVID cohort (South Korea; January 1 to May 15, 2020).

Characteristic				Patients tested for SARS-CoV-2 infection (n=214,207)				Patients with laboratory-confirmed SARS-CoV-2 infection (n=7566)		
				Nonusers of statin		Users of statin		Nonusers of statin		Users of statin
Total, n (%)				178,897 (83.52)		35,310 (16.48)		6547 (86.53)		1019 (13.47)
Age in years, mean (SD)				46.0 (19.0)		67.6 (13.6)		43.5 (17.8)		65.5 (13.7)
**Sex, n (%)**										
	Male		84,477 (47.22)		17,004 (48.16)		2996 (45.76)		490 (48.09)	
	Female		94,420 (52.78)		18,306 (51.84)		3551 (54.24)		529 (51.91)	
**Region of residence, n (%)**										
	Rural		79,786 (44.60)		14,216 (40.26)		2492 (38.06)		423 (41.51)	
	Urban		99,111 (55.40)		21,094 (59.74)		4055 (61.94)		596 (58.49)	
History of cardiovascular disease, n (%)				15,969 (8.93)		14,339 (40.61)		448 (6.84)		343 (33.66)
History of cerebrovascular disease, n (%)				10,745 (6.01)		9582 (27.14)		304 (4.64)		250 (24.53)
History of diabetes mellitus, n (%)				17,949 (10.03)		17,293 (48.97)		559 (8.54)		472 (46.32)
History of chronic obstructive pulmonary disease, n (%)				12,106 (6.77)		5511 (15.61)		368 (5.62)		137 (13.44)
History of hypertension, n (%)				35,166 (19.66)		26,694 (75.60)		1083 (16.54)		729 (71.54)
History of chronic kidney disease, n (%)				7856 (4.39)		6190 (17.53)		372 (5.68)		172 (16.88)
**Charlson Comorbidity** **Index, n (%)**										
		0	116,359 (65.04)		3915 (11.09)		4557 (69.60)		129 (12.66)	
		1	20,728 (11.59)		4418 (12.51)		717 (10.95)		157 (15.41)	
		≥2	41,810 (23.37)		26,977 (76.40)		1273 (19.44)		733 (71.93)	
**Use of medication, n (%)**										
		Aspirin	5502 (3.08)		9066 (25.68)		112 (1.71)		243 (23.85)	
		Metformin	7090 (3.96)		10,664 (30.20)		210 (3.21)		322 (31.60)	
		Systemic glucocorticoids	64,441 (36.02)		14,149 (40.07)		2085 (31.85)		390 (38.27)	

^a^GRCP: Global Research Collaboration Project.

### SARS-CoV-2 Test Positivity and Statins in the Matched GRCP-COVID Cohort

**Figure 2:**
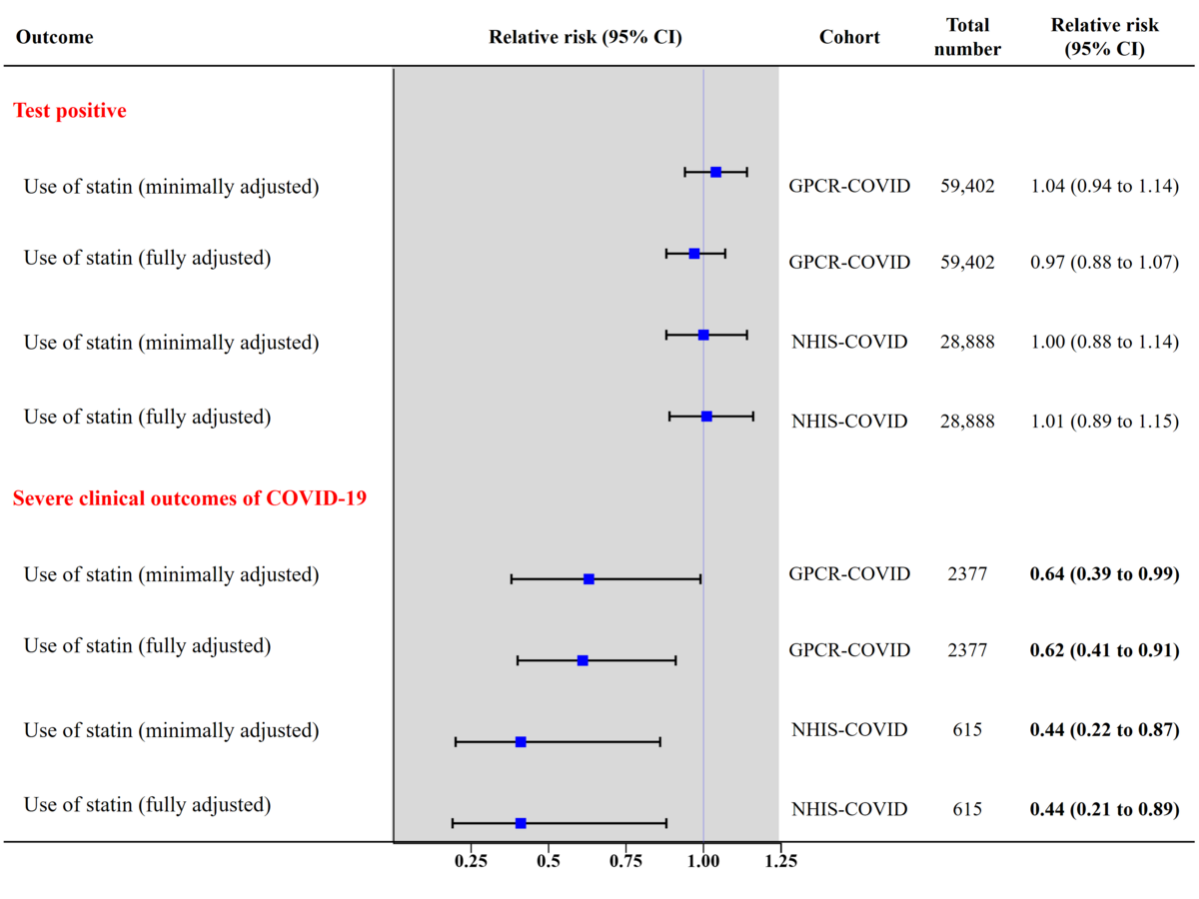
Propensity score-matched association of statin use with (1) positive SARS-CoV-2 test result among patients who underwent SARS-CoV-2 testing (primary outcome), and (2) severe clinical outcomes of COVID-19 among patients who tested positive for SARS-CoV-2 (secondary outcome) in the GPCR-COVID cohort and in the NHIS-COVID cohort (South Korea). Significant values are in bold. GRCP: Global Research Collaboration Project; NHIS: National Health Insurance Service.

After propensity score matching among patients who underwent SARS-CoV-2 testing, we found there were no major imbalances in the baseline covariates between the 2 groups assessed by SMD (Table 2; SMD all <0.1). Among all patients, we identified 29,701 statin users (mean age 67.5 [SD 15.0] years; men, 50.39% [14,966/29,701]) and matched nonusers (mean age, 66.1 [SD 13.8] years; men, 48.89% [14,522/29,701]) in the propensity score–matched cohort. The SARS-CoV-2 test positivity rate was 2.82% (837/29,701) and 2.65% (787/29,701; fully aRR 0.97; 95% CI 0.88-1.07) for statin users and nonusers, respectively (Figure 2).

**Table 2:** Propensity score–matched baseline characteristics, positive SARS-CoV-2 infection test results, and statin use in all patients who were tested for SARS-CoV-2 infection in the GRCP^a^-COVID cohort (n=59,402; South Korea; January 1 to May 15, 2020).

Characteristic			Nonusers of statin (n=29,701)		Users of statin (n=29,701)		Standardized mean difference^b^
Age, years (SD)			67.5 (15.0)		66.1 (13.8)		0.094
**Sex, n (%)**							0.030
	Male	14,966 (50.39)		14,522 (48.89)			
	Female	14,735 (49.61)		15,179 (51.11)			
**Region of residence, n (%)**							0.005
	Rural	12,095 (40.72)		12,031 (40.51)			
	Urban	17,606 (59.28)		17,670 (59.49)			
History of cardiovascular disease, n (%)			10,079 (33.93)		10,660 (35.89)		0.049
History of cerebrovascular disease, n (%)			6988 (23.53)		7210 (24.28)		0.021
History of diabetes mellitus, n (%)			12,176 (41.00)		12,597 (42.41)		0.034
History of chronic obstructive pulmonary disease, n (%)			4733 (15.94)		4557 (15.34)		0.019
History of hypertension, n (%)			22,250 (74.91)		21,180 (71.31)		0.087
History of chronic kidney disease, n (%)			4613 (15.53)		4705 (15.84)		0.010
**Charlson Comorbidity Index, n (%)**							0.015
	0	4664 (15.70)		3859 (12.99)			
	1	4526 (15.24)		4322 (14.55)			
	≥2	20,511 (69.06)		21,520 (72.46)			
**Use of medication, n (%)**							
	Aspirin	4865 (16.38)		5738 (19.32)		0.076	
	Metformin	5835 (19.65)		6938 (23.36)		0.090	
	Systemic glucocorticoids	11,923 (40.14)		11,923 (40.14)		<0.001	
**COVID-19, n (%)^c,d^**			787 (2.65)		837 (2.82)		

^a^GRCP: Global Research Collaboration Project.

^b^A standardized mean difference (SMD) below 0.1 indicates no major imbalance. All SMD values were less than 0.1 in the propensity score–matched cohort.

^c^Minimally adjusted relative risk (95% CI): 1.04 (0.94-1.14), *P*=.43; risk factors were adjusted for age and sex.

^d^Fully adjusted relative risk (95% CI): 0.97 (0.88-1.07), *P*=.55; risk factors were adjusted for age; sex; region of residence (rural or urban); history of diabetes mellitus, cardiovascular disease, cerebrovascular disease, chronic obstructive pulmonary disease, hypertension, or chronic kidney disease; Charlson Comorbidity Index (0, 1, or ≥2); and use of aspirin, metformin, or systemic glucocorticoid.

### Clinical Outcomes in Patients With Laboratory-Confirmed SARS-CoV-2 in the Matched GRCP-COVID Cohort

After propensity score matching among patients who tested positive for SARS-CoV-2, we found there were no major imbalances in the baseline covariates between the 2 groups assessed by SMD (Table 3; SMD all <0.1). Among the patients with laboratory-confirmed COVID-19, the severe clinical outcomes were 3.98% (32/804) and 5.40% (85/1573) for statin users and nonusers, respectively (Figure 2; fully aRR 0.62; 95% CI 0.41-0.91). Moreover, statin users were hospitalized for an average of 23.8 days compared with 26.3 days for nonusers (adjusted mean difference –2.87; 95% CI –5.68 to –0.93).

**Table 3:** Propensity score–matched baseline characteristics and the composite endpoint with statin use among patients with laboratory-confirmed SARS-CoV-2 infection in the GRCP^a^-COVID cohort (n=2377; South Korea; January 1 to May 15, 2020).

Characteristic		Nonusers of statin (n=1573)	Users of statin (n=804)	Standardized mean difference^b^
Age, years (SD)		63.6 (15.7)	63.7 (13.9)	0.003
**Sex, n (%)**				0.009
	Male	745 (47.4)	377 (46.9)	
	Female	828 (52.6)	427 (53.1)	
**Region of residence, n (%)**				0.006
	Rural	647 (41.1)	333 (41.4)	
	Urban	926 (58.9)	471 (58.6)	
History of cardiovascular disease, n (%)		410 (26.1)	229 (28.5)	0.054
History of cerebrovascular disease, n (%)		259 (16.5)	163 (20.3)	0.098
History of diabetes mellitus, n (%)		501 (31.8)	294 (36.6)	0.100
History of chronic obstructive pulmonary disease, n (%)		198 (12.6)	103 (12.8)	0.008
History of hypertension, n (%)		957 (60.8)	525 (65.3)	0.093
History of chronic kidney disease, n (%)		263 (16.7)	133 (16.5)	0.006
**Charlson Comorbidity** **Index, n (%)**				0.099
	0	489 (31.1)	122 (15.2)	
	1	203 (12.9)	142 (17.7)	
	≥2	881 (56.0)	540 (67.2)	
**Use of medication, n (%)**				
	Aspirin	105 (6.7)	75 (9.3)	0.099
	Metformin	194 (12.3)	123 (15.3)	0.087
	Systemic glucocorticoids	622 (39.5)	305 (37.9)	0.034
Severe outcomes of COVID-19^c,d,e^, n (%)		85 (5.4)^g^	32 (4.0)^g^	
Length of stay for patients in hospital (days), mean (SD)^f^		26.3 (15.7)^g^	23.8 (14.2)^g^	

^a^GRCP: Global Research Collaboration Project.

^b^A standardized mean difference (SMD) below 0.1 indicates no major imbalance. All SMD values were less than 0.1 in the propensity score–matched cohort.

^c^Severe outcomes of COVID-19 consisted of admission to the intensive care unit, invasive ventilation, or death.

^d^Minimally adjusted relative risk (95% CI): 0.64 (0.40 to 0.96), *P*=.04; risk factors were adjusted for age and sex.

^e^Fully adjusted relative risk (95% CI): 0.62 (0.41 to 0.91), *P*=.02; risk factors were adjusted for age; sex; region of residence (rural or urban); histories of diabetes mellitus, cardiovascular disease, cerebrovascular disease, chronic obstructive pulmonary disease, hypertension, or chronic kidney disease; and Charlson Comorbidity Index (0, 1, or ≥2); and use of aspirin, metformin, or systemic glucocorticoid.

^f^The fully adjusted mean difference (95% CI) was –2.87 (–5.68 to –0.93). Risk factors were adjusted for age and sex.

^g^Statistically significant differences (*P*<.05).

### Unmatched NHIS-COVID Cohort

**Figure 3:**
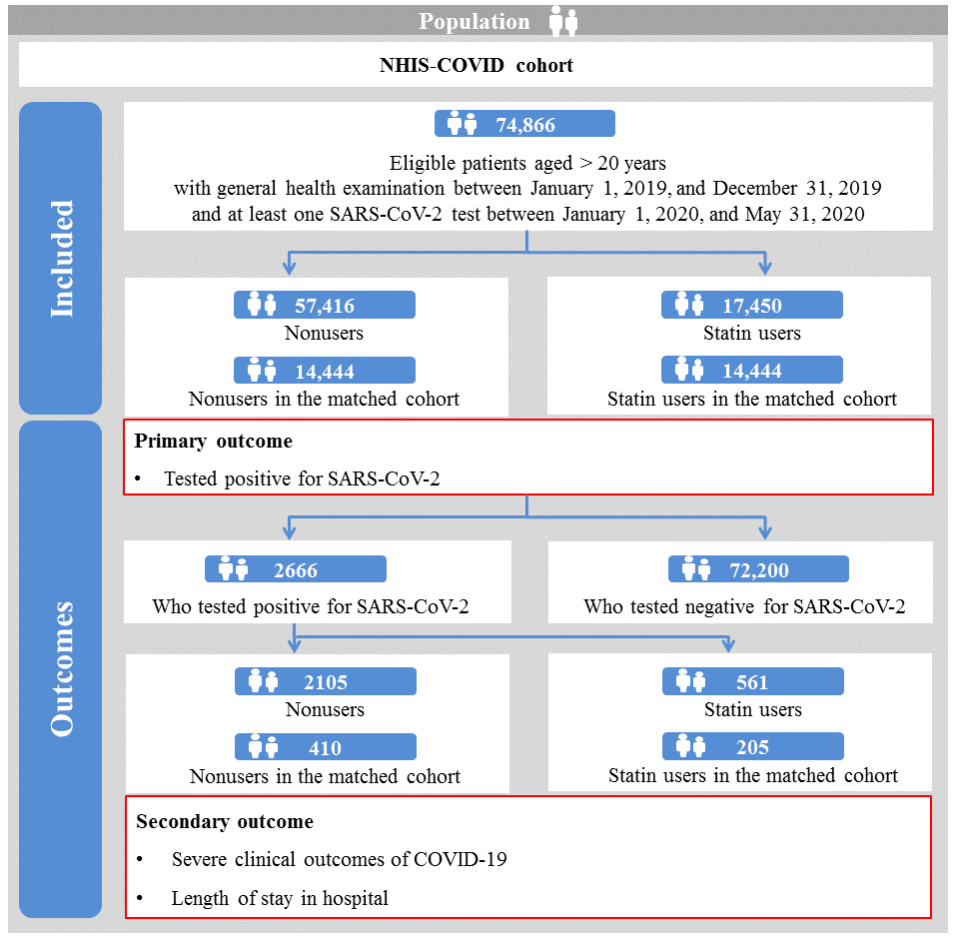
Graphical depiction of patient enrollment in the NHIS-COVID cohort (South Korea; January 1 to May 31, 2020). NHIS: National Health Insurance Service.

Among patients who underwent SARS-CoV-2 testing (n=74,866), we identified 57,416 nonusers and 17,450 statin users in the full unmatched cohort (Table 4 and Figure 3 and Multimedia Appendix 1). Among patients with laboratory-confirmed COVID-19 (n=2666), we identified 2105 nonusers and 561 statin users in the full unmatched cohort.

**Table 4:** Baseline characteristics of all patients who were tested for SARS-CoV-2 infection and of those with laboratory-confirmed SARS-CoV-2 infection in the NHIS^a^-COVID cohort (South Korea; January 1 to May 31, 2020).

Characteristics			Patients tested for SARS-CoV-2 infection (n=74,866)				Patients with laboratory-confirmed SARS-CoV-2 infection (n=2666)		
			Users of statin		Nonusers of statin		Users of statin		Nonusers of statin
Total, n (%)			57,416 (76.69)		17,450 (23.3)		2105 (79.0)		561 (21.0)
**Age (years), n (%)**									
	20-59	38,120 (66.39)		4443 (25.46)		1401 (66.56)		169 (30.12)	
	60-69	7987 (13.91)		4721 (27.05)		422 (20.05)		187 (33.33)	
	≥70	11,309 (19.70)		8286 (47.48)		282 (13.40)		205 (36.54)	
**Sex, n (%)**									
	Male	28,092 (48.93)		8289 (47.50)		770 (36.58)		202 (36.01)	
	Female	29,324 (51.07)		9161 (52.50)		1335 (63.42)		359 (63.99)	
**Region of residence, n (%)**									
	Rural	20,892 (36.39)		6385 (36.59)		216 (10.26)		39 (6.95)	
	Urban	36,524 (63.61)		11,065 (63.41)		1889 (89.74)		522 (93.05)	
History of diabetes mellitus, n (%)			3603 (6.28)		5227 (29.95)		103 (4.89)		169 (30.12)
History of stroke, n (%)			694 (1.21)		881 (5.05)		13 (0.62)		22 (3.92)
History of cardiovascular disease, n (%)			1215 (2.12)		2531 (14.50)		25 (1.19)		52 (9.27)
**Charlson Comorbidity Index, n (%)**									
	0	37,320 (65.00)		1939 (11.11)		1201 (57.05)		56 (9.98)	
	1	6660 (11.60)		2181 (12.50)		362 (17.20)		100 (17.83)	
	≥2	13,436 (23.40)		13,330 (76.39)		542 (25.75)		405 (72.19)	
**BMI (kg/m^2^), n (%)**									
	<25	38,976 (67.88)		9467 (54.25)		1427 (67.79)		309 (55.08)	
	25-30	15,524 (27.04)		6652 (38.12)		587 (27.89)		223 (39.75)	
	>30	2916 (5.08)		1331 (7.63)		91 (4.32)		29 (5.17)	
Systolic blood pressure (mmHg), mean (SD)			121.4 (15.2)		128.2 (15.5)		120.4 (15.2)		127.6 (15.4)
Diastolic blood pressure (mmHg), mean (SD)			74.9 (10.1)		76.8 (10.2)		74.4 (10.1)		77.0 (10.1)
Fasting blood glucose (mg/dL), mean (SD)			98.6 (24.7)		112.8 (37.4)		98.5 (25.6)		112.7 (33.5)
Serum total cholesterol (mg/dL), mean (SD)			191.5 (37.1)		182.4 (50.0)		195.3 (35.7)		188.3 (46.5)
Serum low-density lipoprotein cholesterol (mg/dL), mean (SD)			110.8 (33.0)		101.5 (45.5)		115.3 (31.5)		106.6 (42.0)
Serum high-density lipoprotein cholesterol (mg/dL), mean (SD)			56.9 (18.8)		53.5 (16.5)		57.9 (27.3)		56.5 (38.4)
**Estimated glomerular filtration rate (mL/min/1.73m^2^), n (%)**									
	Normal (≥90)	29,258 (51.96)		5693 (32.62)		1066 (50.64)		216 (38.50)	
	Mildly decreased (60-89)	24,714 (43.04)		8962 (51.36)		958 (45.51)		289 (51.52)	
	Moderately to severely decreased (<59)	3444 (6.00)		2795 (16.02)		81 (3.85)		56 (9.98)	
**Household income, n (%)**									
	Low (0th-39th percentile)	15,480 (27.96)		4771 (27.34)		784 (37.24)		185 (32.98)	
	Middle (40th-79th percentile)	21,313 (37.12)		5227 (29.95)		685 (32.54)		178 (31.73)	
	High (80th-100th percentile)	20,623 (35.92)		7452 (42.70)		636 (30.21)		198 (35.29)	
**Smoking, n (%)**									
	Never smoker	37,145 (64.69)		11,270 (64.58)		1681 (79.86)		419 (74.69)	
	Ex-smoker	9278 (16.16)		3708 (21.25)		280 (13.30)		101 (18.00)	
	Current smoker	10,993 (19.15)		2472 (14.17)		144 (6.84)		41 (7.31)	
**Alcoholic drinks per week, n (%)**									
	<1	32,184 (56.05)		12,667 (72.59)		1472 (69.93)		423 (75.40)	
	1-2	18,048 (31.43)		3152 (18.06)		489 (23.23)		94 (16.76)	
	3-4	4981 (8.68)		1021 (5.85)		103 (4.89)		32 (5.70)	
	≥5	2203 (3.84)		610 (3.50)		41 (1.95)		12 (2.14)	
**Physical activity sessions per week, n (%)**									
	0	29,958 (52.18)		10,061 (57.66)		1154 (54.82)		331 (59.00)	
	1-2	14,079 (24.52)		2943 (16.87)		469 (22.28)		86 (15.33)	
	3-4	7563 (13.17)		2301 (13.19)		286 (13.59)		73 (13.01)	
	5-6	3846 (6.70)		1253 (7.18)		141 (6.70)		39 (6.95)	
	7	1970 (3.43)		892 (5.11)		55 (2.61)		32 (5.70)	
**Use of medication, n (%)**									
	Medication for hypertension, n (%)	9074 (15.80)		8939 (51.23)		266 (12.64)		263 (46.88)	
	Medication for diabetes mellitus, n (%)	3328 (5.80)		4989 (28.59)		93 (4.42)		159 (28.34)	
	Medication for cardiovascular disease, n (%)	1028 (1.79)		2400 (13.75)		18 (0.86)		52 (9.27)	

^a^NHIS: National Health Insurance Service.

### SARS-CoV-2 Test Positivity and Statins in the Matched NHIS-COVID Cohort

After propensity score matching among patients who underwent SARS-CoV-2 testing, we found there were no major imbalances in the baseline covariates between 2 groups assessed by SMD (Table 5; SMD all <0.1). Among all patients, we identified 14,444 statin users and matched nonusers in the propensity score–matched cohort. The SARS-CoV-2 test positivity rate was 3.3% (483/14,444) and 3.4% (492/14,444; fully aRR 1.01; 95% CI 0.89-1.15) for statin users and nonusers, respectively (Figure 2).

**Table 5:** Propensity score–matched baseline characteristics, positive SARS-CoV-2 infection test results, and statin use in all patients who were tested for SARS-CoV-2 infection in the NHIS^a^-COVID cohort (n=28,888; South Korea; January 1 to May 31, 2020).

Characteristic		Nonusers of statins	Users of statins	Standardized mean difference^b^
Total, n		14,444	14,444	
**Age (years), n (%)**				0.099
	20-59	4524 (31.32)	4479 (31.01)	
	60-69	2938 (20.34)	3505 (24.27)	
	≥70	6982 (48.34)	6460 (44.72)	
**Sex, n (%)**				0.019
	Male	7107 (49.20)	6967 (48.23)	
	Female	7337 (50.80)	7477 (51.77)	
**Region of residence, n (%)**				0.009
	Rural	5341 (36.98)	5277 (36.53)	
	Urban	9103 (63.02)	9167 (63.47)	
History of diabetes mellitus, n (%)		3002 (20.78)	3434 (23.77)	0.082
History of stroke, n (%)		537 (3.72)	580 (4.02)	0.017
History of cardiovascular disease, n (%)		1013 (7.01)	1367 (9.46)	0.089
**Charlson Comorbidity** **Index, n (%)**				0.049
	0	2549 (17.65)	1341 (9.28)	
	1	1524 (10.55)	1879 (13.01)	
	≥2	10,371 (71.80)	11,224 (77.71)	
**Body mass index (kg/m^2^), n (%)**				0.049
	<25	7966 (55.15)	8154 (56.45)	
	25-30	5167 (35.77)	5331 (36.91)	
	>30	1311 (9.08)	959 (6.64)	
Systolic blood pressure (mmHg), mean (SD)		128.5 (16.0)	127.3 (15.3)	0.082
Diastolic blood pressure (mmHg), mean (SD)		77.4 (10.4)	76.6 (10.1)	0.075
Fasting blood glucose (mg/dL), mean (SD)		109.5 (38.2)	109.9 (34.2)	0.013
Serum total cholesterol (mg/dL), mean (SD)		189.7 (38.9)	186.0 (50.9)	0.083
Serum low-density lipoprotein cholesterol (mg/dL), mean (SD)		108.4 (33.5)	105.0 (46.7)	0.088
Serum high-density lipoprotein cholesterol (mg/dL), mean (SD)		53.5 (15.5)	54.0 (17.0)	0.025
**Estimated glomerular filtration rate (mL/min/1.73 m^2^), n (%)**				0.018
	Normal (≥90)	5214 (36.10)	5018 (34.74)	
	Mildly decreased (60-89)	6976 (48.30)	7468 (51.70)	
	Moderately to severely decreased (<59)	2254 (15.61)	1958 (13.56)	
**Household income, n (%)**				0.005
	Low (0th-39th percentile)	3802 (26.32)	4002 (27.71)	
	Middle (40th-79th percentile)	4688 (32.46)	4368 (30.24)	
	High (80th-100th percentile)	5954 (41.22)	6074 (42.05)	
**Smoking, n (%)**				0.007
	Never smoker	9319 (64.52)	9250 (64.04)	
	Ex-smoker	2845 (19.70)	3058 (21.17)	
	Current smoker	2280 (15.79)	2136 (14.79)	
**Alcoholic drinks per week, n (%)**				0.002
	<1	10,174 (70.44)	10,192 (70.56)	
	1-2	2817 (19.50)	2794 (19.34)	
	3-4	909 (6.29)	918 (6.36)	
	≥5	544 (3.77)	540 (3.74)	
**Physical activity sessions per week, n (%)**				0.007
	0	8268 (57.24)	8241 (57.05)	
	1-2	2555 (17.69)	2541 (17.59)	
	3-4	1781 (12.33)	1917 (13.27)	
	5-6	1066 (7.38)	1063 (7.36)	
	7	774 (5.36)	682 (4.72)	
**Use of medication, n (%)**				
	Medication for hypertension, n (%)	6758 (46.79)	6515 (45.11)	0.038
	Medication for diabetes mellitus, n (%)	2797 (19.36)	3246 (22.47)	0.086
	Medication for cardiovascular disease, n (%)	904 (6.26)	1280 (8.86)	0.099
COVID-19, n (%)^c,d^		483 (3.34)	492 (3.41)	

^a^NHIS: National Health Insurance Service.

^b^A standardized mean difference (SMD) of less than 0.1 indicates no major imbalance. All SMD values were less than 0.1 in each propensity score–matched cohort.

^c^Minimally adjusted relative risk (95% CI): 1.00 (0.88-1.13), *P*=.99; risk factors were adjusted for age and sex.

^d^Fully adjusted relative risk (95% CI): 1.01 (0.89-1.15), *P*=.89; risk factors were adjusted for age; sex; region of residence (rural and urban); history of diabetes mellitus, stroke, cardiovascular disease; Charlson Comorbidity Index (0, 1, or ≥2); body mass index (<25, 25-30, and ≥30 kg/m^2^); systolic blood pressure (continuous); diastolic blood pressure (continuous); fasting blood glucose (continuous); serum total cholesterol (continuous); serum low-density lipoprotein (continuous); serum high-density lipoprotein (continuous); estimated glomerular filtration rate (normal, mildly decreased, and moderately to severely decreased); household income (low, middle, and high); smoking (never smoker, ex-smoker, and current smoker); frequency of alcohol consumption (<1, 1-2, 3-4, 5-6, and 7 times per week); physical activity (0, 1-2, 3-4, 5-6, and 7 sessions per week); and medication for hypertension, diabetes mellitus, and cardiovascular disease.

### Clinical Outcomes in Patients With Laboratory-Confirmed SARS-CoV-2 in the Matched NHIS-COVID Cohort

After propensity score matching among patients who tested positive for SARS-CoV-2, we found there were no major imbalances in the baseline covariates between the 2 groups assessed by SMD (Table 6; SMD all <0.1), except medication for cardiovascular disease among nonusers versus statin users (SMD 0.120). Among the patients with laboratory-confirmed COVID-19, the severe clinical outcomes were 5.4% (11/205) and 12.2% (50/410) for statin users and nonusers, respectively (Figure 2; fully aRR 0.44; 95% CI 0.21-0.89). Moreover, statin users were hospitalized for an average of 23.9 days compared with 26.3 days for nonusers (adjusted mean difference –2.53; 95% CI –5.54 to –0.37).

**Table 6:** Propensity score–matched baseline characteristics and the severe clinical outcomes with statin use among patients with laboratory-confirmed SARS-CoV-2 infection in the NHIS^a^-COVID cohort (n=615; South Korea; January 1 to May 31, 2020).

Characteristic		Nonusers of statins (n=410)	Users of statins (n=205)	Standardized mean difference^b^
**Age (years), n (%)**				0.011
	20-59	175 (42.7)	88 (42.9)	
	60-69	121 (29.5)	61 (29.8)	
	≥70	114 (27.8)	56 (27.3)	
**Sex, n (%)**				0.081
	Male	132 (32.2)	74 (36.1)	
	Female	278 (67.8)	131 (63.9)	
**Region of residence, n (%)**				0.043
	Rural	39 (9.5)	17 (8.3)	
	Urban	371 (90.5)	188 (91.7)	
History of diabetes mellitus, n (%)		40 (9.8)	24 (11.7)	0.063
History of stroke, n (%)		4 (1.0)	3 (1.5)	0.044
History of cardiovascular disease, n (%)		8 (2.0)	7 (3.4)	0.091
**Charlson Comorbidity Index** **, n (%)**				0.077
	0	98 (23.9)	42 (20.5)	
	1	107 (26.1)	59 (28.8)	
	≥2	208 (50.7)	104 (50.7)	
**Body mass index (kg/m^2^), n (%)**				0.090
	<25	251 (61.2)	128 (62.4)	
	25-30	140 (34.1)	71 (34.6)	
	>30	19 (4.6)	6 (2.9)	
Systolic blood pressure (mmHg), mean (SD)		125.1 (15.6)	123.7 (15.5)	0.090
Diastolic blood pressure (mmHg), mean (SD)		77.2 (10.4)	76.3 (10.4)	0.086
Fasting blood glucose (mg/dL), mean (SD)		105.6 (29.4)	102.8 (20.6)	0.093
Serum total cholesterol (mg/dL), mean (SD)		201.0 (37.8)	203.0 (49.5)	0.050
Serum low-density lipoprotein cholesterol (mg/dL), mean (SD)		117.9 (33.3)	121.5 (43.7)	0.093
Serum high-density lipoprotein cholesterol (mg/dL), mean (SD)		58.2 (16.8)	58.2 (52.1)	<0.001
**Estimated glomerular filtration rate (mL/min/1.73 m^2^), n (%)**				0.067
	Normal (≥90)	171 (41.7)	81 (39.5)	
	Mildly decreased (60-89)	207 (50.5)	110 (53.7)	
	Moderately to severely decreased (<59)	32 (7.8)	14 (6.8)	
**Household income, n (%)**				0.096
	Low (0th-39th percentile)	141 (34.4)	68 (33.2)	
	Middle (40th-79th percentile)	141 (34.4)	64 (31.2)	
	High (80th-100th percentile)	128 (31.2)	73 (35.6)	
**Smoking, n (%)**				0.084
	Never smoker	318 (77.6)	155 (75.6)	
	Ex-smoker	66 (16.1)	39 (19.0)	
	Current smoker	26 (6.3)	11 (5.4)	
**Alcoholic drinks per week, n (%)**				0.080
	<1	314 (76.6)	153 (74.6)	
	1-2	70 (17.1)	39 (19.0)	
	3-4	19 (4.6)	8 (3.9)	
	≥5	7 (1.7)	5 (2.4)	
**Physical activity sessions per week, n (%)**				0.085
	0	233 (56.8)	118 (57.6)	
	1-2	85 (20.7)	37 (18.0)	
	3-4	59 (14.4)	34 (16.6)	
	5-6	22 (5.4)	11 (5.4)	
	7	11 (2.7)	5 (2.4)	
**Use of medication, n (%)**				
	Medication for hypertension, n (%)	102 (24.9)	46 (22.4)	0.057
	Medication for diabetes mellitus, n (%)	36 (8.8)	22 (10.7)	0.066
	Medication for cardiovascular disease, n (%)	5 (1.2)	6 (2.9)	0.120
Severe clinical outcomes of COVID-19, n (%)^c,^^d^		50 (12.2)^e^	11 (5.4)^e^	
Length of stay for patients in hospital (days), mean (SD)		26.3 (15.7)^e^	23.9 (14.3)^e^	

^a^NHIS: National Health Insurance Service.

^b^A standardized mean difference (SMD) below 0.1 indicates no major imbalance. All SMD values were less than 0.1 in the propensity score–matched cohort, except medication for cardiovascular disease among nonusers versus statin users.

^c^Minimally adjusted relative risk (95% CI): 0.44 (0.22-0.87), *P*=.02; risk factors were adjusted for age and sex.

^d^Fully adjusted relative risk (95% CI): –2.53 (–5.54 to –0.37); *P*=.03; risk factors were adjusted for age; sex; region of residence (rural and urban); history of diabetes mellitus, stroke, cardiovascular disease; Charlson Comorbidity Index (0, 1, or ≥2); body mass index (<25, 25-30, and ≥30 kg/m^2^); systolic blood pressure (continuous); diastolic blood pressure (continuous); fasting blood glucose (continuous); serum total cholesterol (continuous); serum low-density lipoprotein (continuous); serum high-density lipoprotein (continuous); estimated glomerular filtration rate (normal, mildly decreased, and moderately to severely decreased); household income (low, middle, and high); smoking (never smoker, ex-smoker, and current smoker); frequency of alcohol consumption (<1, 1-2, 3-4, 5-6, and 7 times per week); physical activity (0, 1-2, 3-4, 5-6, and 7 sessions per week); and medication for hypertension, diabetes mellitus, and cardiovascular disease.

^e^Statistically significant differences (*P*<.05).

## Discussion

### Principal Findings

Among those in the GRCP-COVID cohort (n=214,207) and NHIS-COVID cohort (n=74,866) who underwent SARS-CoV-2 testing, 16.5% (35,310/214,207) and 23.3% (17,450/74,866) were currently taking statins, respectively. We examined the potential association between positive SARS-CoV-2 test results with the current use of statins in the propensity score–matched cohort (GRCP-COVID, n=59,402; NHIS-COVID, n=28,888) and clinical outcomes of patients with COVID-19 taking statins in the propensity score–matched cohort (GRCP-COVID, n=2377; NHIS-COVID, n=615). After controlling for various confounding variables using propensity matching and statistical adjustment, the use of statins was associated with improved clinical outcomes of COVID-19 and decreased length of hospital stay but not with the risk of susceptibility to SARS-CoV-2 infection.

This study demonstrated that statin use was associated with improved clinical outcomes and decreased length of hospital stay in patients with COVID-19. Many plausible pathophysiology could contribute to the association of statin use with COVID-19 outcomes.

The entry of SARS-CoV-2 is initiated by the binding of viral spike protein to the cellular receptor ACE2 [26]. The intracellular invasion of SARS-CoV-2 via ACE2 downregulates the expression of ACE2, which results in disinhibition of angiotensin II [27]. This, in turn, induces vasoconstriction of lung vasculatures as well as increases vascular permeability and inflammation, thereby leading to lung injury [28]. Statins are known to upregulate ACE2, and thus are recognized as promising antiviral agents [11].

Immune modulatory effects of statins may influence recovery from COVID-19. COVID-19 is accompanied by the activation of the immune system and the elevation of inflammatory cytokines, such as C-reactive protein, ferritin, and interleukin-6 (IL-6) [29], and could induce a severe catastrophe response with hyperinflammation, namely, the “cytokine storm” [30]. Thus, controlling host response and restoring immune homeostasis may be crucial to reduce the severity of COVID-19. In this context, the control of host response using immune modulation is a promising therapeutic option for SARS-CoV-2 infection [31]. By affecting chemokine secretion and expression of adhesion molecules, such as lymphocyte function–associated antigen 1 and intercellular adhesion molecule 1, statins could modulate immune cell trafficking to the airways [32]. Moreover, statins could suppress the activation of Th1 cells and interferon-γ (a key player of the chronic inflammation) [33], and downregulate major histocompatibility complex class II expression in the airway including B cells [31]. Therefore, statins were reported to be effective in treating immune disorders, such as multiple sclerosis [34] and rheumatoid arthritis [35]. This evidence of widespread anti-inflammatory effects implies that long-term use of statins could act as a shield to protect patients from severe cascades of inflammation represented by the cytokine storm [11].

Statins are a well-known class of drugs that protect the vascular endothelium from reactive oxygen species [36]. A major effect of statins on endothelial cells is the decreased number of reactive oxygen species due to the downregulation of endothelin-1 expression, and decreased proinflammatory cytokines (eg, IL-1β, IL-6) [36]. Additionally, statins are known to suppress the expression of caveolin-1, a key component of endothelial cell transcytosis [37], which may ameliorate the vascular hyperpermeability induced by the inflammatory manifestations of SARS-CoV-2 infection. These vasoprotective effects of statins may prevent pulmonary edema and other lung injuries that may eventually lead to acute respiratory distress syndrome, a major cause of death in COVID-19.

Numerous studies have reported the effects of statins on clinical improvements in viral infections, namely, Ebola (positive association) [38], influenza (no association) [39], pneumonia (positive association [40] and no association [41,42]), acute respiratory distress syndrome (no association in the entire group [43], but positive association in a subgroup analysis, namely, a hyperinflammatory subphenotype [44]), hepatitis (positive association [45]), and hepatocellular carcinoma in patients with hepatitis (positive association [23]). However, previous studies on the association of statins with the clinical outcomes of pneumonia were limited by limited sample size [42], restricted clinical outcomes (mortality in ventilator-associated patients with pneumonia [41] or elevation of cytokine levels [42]), or by a focus on specific statin types [41,42].

A recent previous study reported the beneficial association between in-hospital use of statin and mortality in patients with COVID-19 [15], which is consistent with our results. However, unlike our study, this previous study did not analyze statin use before SARS-CoV-2 infection contraction.

### Limitations

This study has some limitations. First, the GRCP-COVID cohort had a maximum follow-up duration less than 3 years, whereas the history of statin use can span decades. For rapid data acquisition and real-time analysis in this global crisis, the Korean Government provides relatively short-term histories (maximum 3 years) of patients with COVID-19. Second, in the GRCP-COVID cohort, the serum lipid profiles of patients were not accessible. However, the Korean National Health Insurance program has strict regulations regarding reimbursement for the treatment of hyperlipidemia that count the risk factors and consider the serum cholesterol levels. This makes the inclusion of the patients with dyslipidemia very robust. Third, important missing covariates included smoking status, blood pressure levels, biomarkers including basal sugar levels, and factors that could influence the outcomes of COVID-19. To overcome this issue, we adjusted the statistics by obtaining the histories of hypertension, diabetes, and COPD, a well-known smoking-associated disorder, verified by ICD codes. Fourth, our analysis lacked the evaluation of patient adherence to statins based on either the medical record or the questionnaire. Further meticulous review of data including the drug adherence is necessary to overcome this issue. Fifth, our analysis lacked personal information of the socioeconomic status (education level and household income) of the patients, which might affect the treatment compliance of patients with COVID-19. However, all COVID-19–related costs were provided complimentarily by the Korean Government; thus, the impact of the socioeconomic status of the patients on the clinical outcomes of COVID-19 may be minimal. Sixth, we were unable to obtain full information about all baseline variables on the index date in the NHIS-COVID cohort; therefore, the values of time-varying variables might differ from their values on the index date. To overcome this issue, we used the data measured on the date closest to the index date as the baseline data. Besides, we did not investigate whether statin medication was continued while the patients were hospitalized. A further study is warranted to clarify this issue. Seventh, as our study cohort involves patients tested for SARS-CoV-2, there was potential for ascertainment bias wherein those who have pre-existing health conditions (some necessitating statins) may have greater COVID-19 awareness, and be more likely to undergo screening, and be referred to care settings. However, the prevalence of patients with statin use (GRCP-COVID cohort, 16.48% [35,310/214,207]) is comparable to a nationally representative cohort of the general US population aged over 20 years (17.23%) [46]. Furthermore, we performed meticulous adjustment of systemic factors by using double-propensity score matching and fully adjusted regression analysis to overcome prevalence-related bias in the group comparisons. Eighth, the possibility of selection bias during the propensity matching process cannot be excluded. Because statins are one of the popular drugs for chronic diseases, such as cardiovascular disease, cerebrovascular disease, and diabetes mellitus, matching on these covariates could select control participants who had similar medical profiles but were not prescribed statins. Although similar medical profiles between nonusers and statin users reveal the direct effect of statin usage, the possibility of selection bias remains. Lastly, every patient involved in this study was Asian in ethnicity. Therefore, our results may not be extrapolatable to different ethnicities. However, the effect of statin use was reported to be effective regardless of the ethnic background [47]. Further studies of large cohorts involving diverse populations are warranted to clarify this issue.

Another limitation was that although the NHIS-COVID cohort had more key covariates than the GRCP cohort (ie, serum glucose, lipid profile, body mass index, household income, smoking status, physical activity status, and frequency of alcohol consumption), the total sample size (n=74,866) was less than that of the GRCP-COVID cohort (n=214,207). Finally, although 2 organizations (NHIS and Health Insurance Review and Assessment Service [HIRA]) independently constructed the cohorts, there is a possibility that patient data may have been duplicated.

### Conclusions

Statins are inexpensive and readily available therapeutic agents. Despite the aforesaid limitations, our study highlights for the first time the potential protective effects of previous statin use on the clinical outcomes of patients with COVID-19. Two large cohort studies provided clinical evidence that previous and current statin use is associated with decreased risks of severe COVID-19 outcomes; one study used claims-based data (GRCP-COVID cohort) and the other used interview-based data (NHIS-COVID cohort), with propensity score matching. Furthermore, our study included a large number of patients and well-designed statistical techniques were implemented. Thus, our use of 2 independent cohorts increases the generalizability and reliability of our results, and this makes the results of this study robust and reliable.

Prior statin use was associated with a decreased likelihood of severe clinical outcomes of COVID-19 and a shorter length of hospital stay. Our well-designed observational study suggests that the use of statins may play a potential protective role for patients with COVID-19 and that randomized controlled trials of the therapeutic use of statins for COVID-19 are warranted.

## Appendix

Multimedia Appendix 1 Supplementary figures.

## Abbreviations

ACE2: angiotensin-converting enzyme 2
aRR: adjusted relative risk
BCG: bacillus Calmette–Guérin
COPD: chronic obstructive pulmonary disease
GRCP: Global Research Collaboration Project
HIRA: Health Insurance Review and Assessment Service
ICD-10: International Classification of Disease 10th revision
IL: interleukin
NHIS: National Health Insurance Service
SMD: standardized mean difference
WHO: World Health Organization
