# Supplementary material for: Statin Use and COVID-19 Infectivity and Severity in South Korea: Two Population-Based Nationwide Cohort Studies
Source: JMIR Public Health Surveill. 2021 Oct 8;7(10):e29379. doi: 10.2196/29379 (PMC8510150; doi:10.2196/29379)

**Figure S1.** Density of propensity scores before and after matching, among all patients who underwent SARS-CoV-2 testing in the GPCR-COVID cohort (n = 59,402).


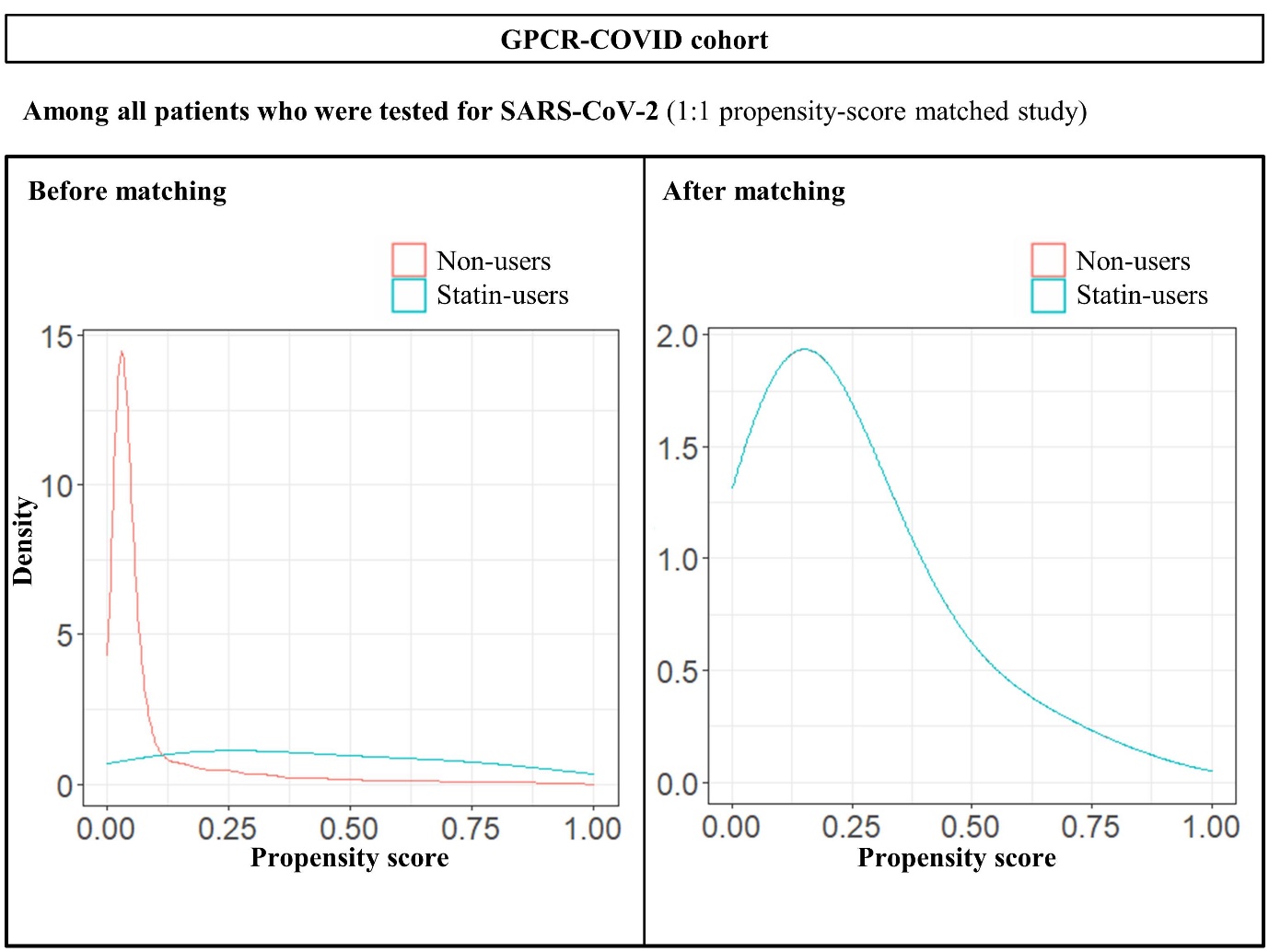


**Figure S2.** Density of propensity scores before and after matching, among all patients who tested positive for SARS-CoV-2 in the GPCR-COVID cohort (n = 2377).


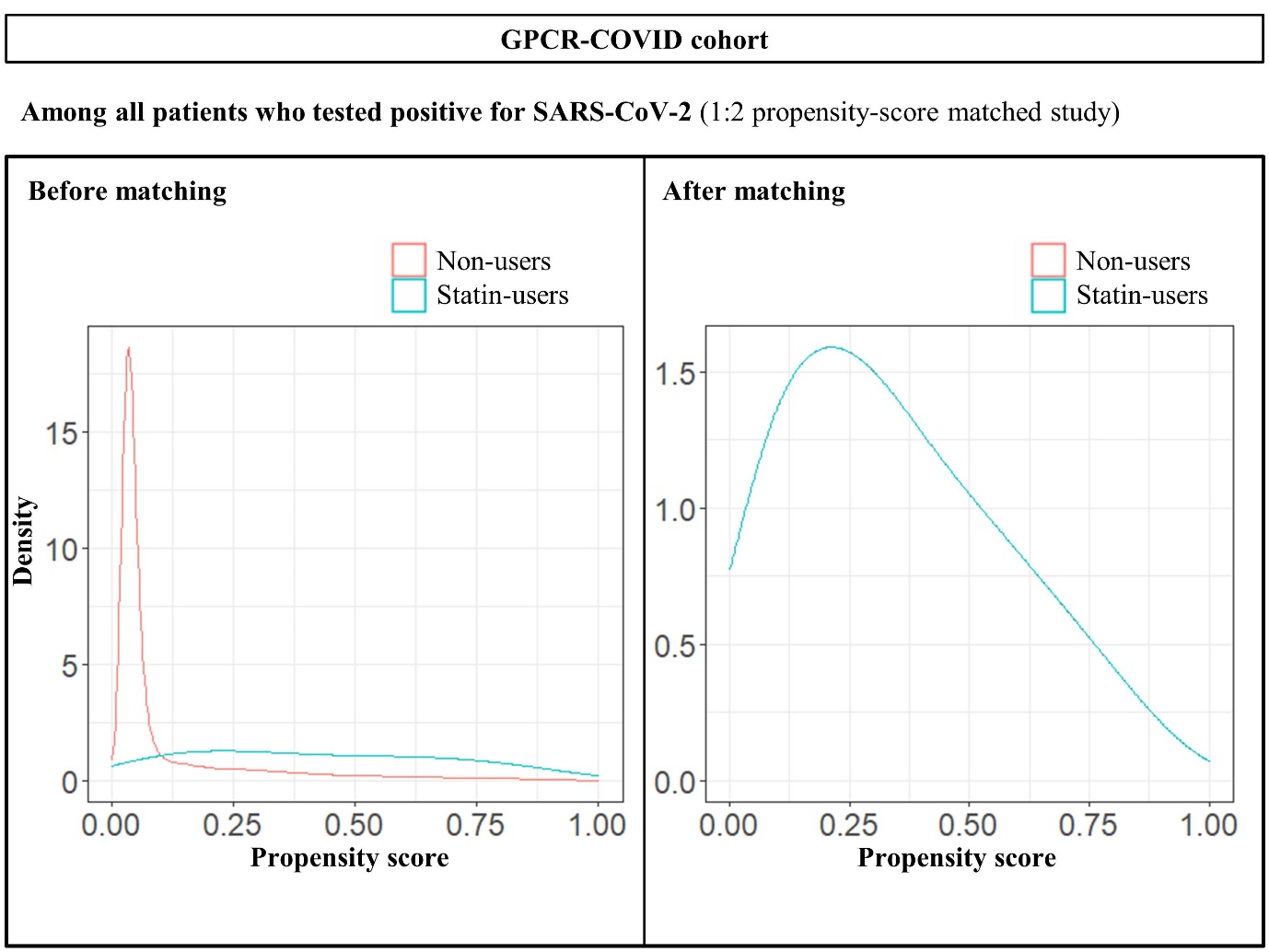


**Figure S3.** Density of propensity scores before and after matching, among all patients who underwent SARS-CoV-2 testing in the NHIS-COVID cohort (n = 28,888).


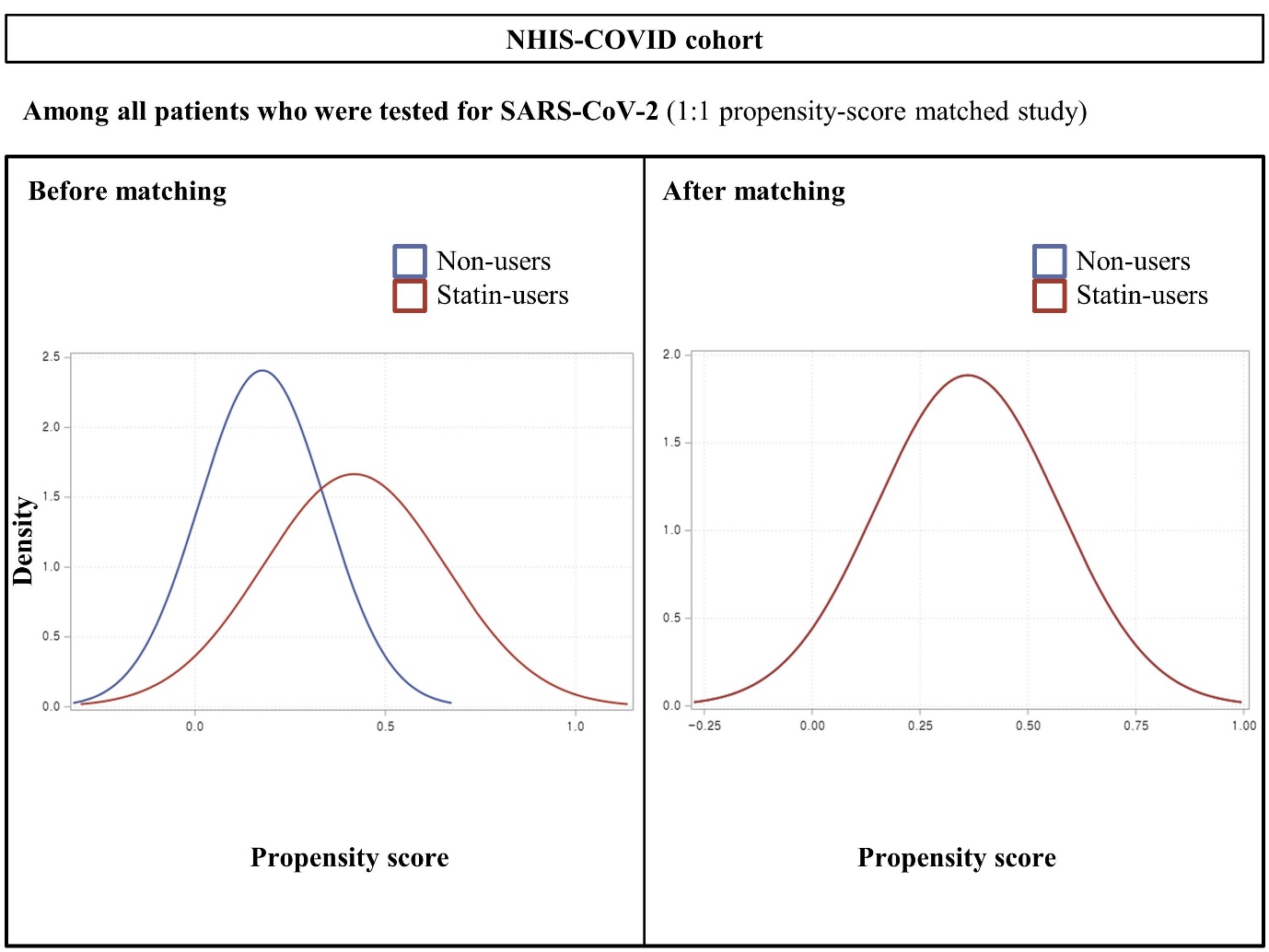


**Figure S4.** Density of propensity scores before and after matching, among all patients who tested positive for SARS-CoV-2 in the NHIS-COVID cohort (n = 615).


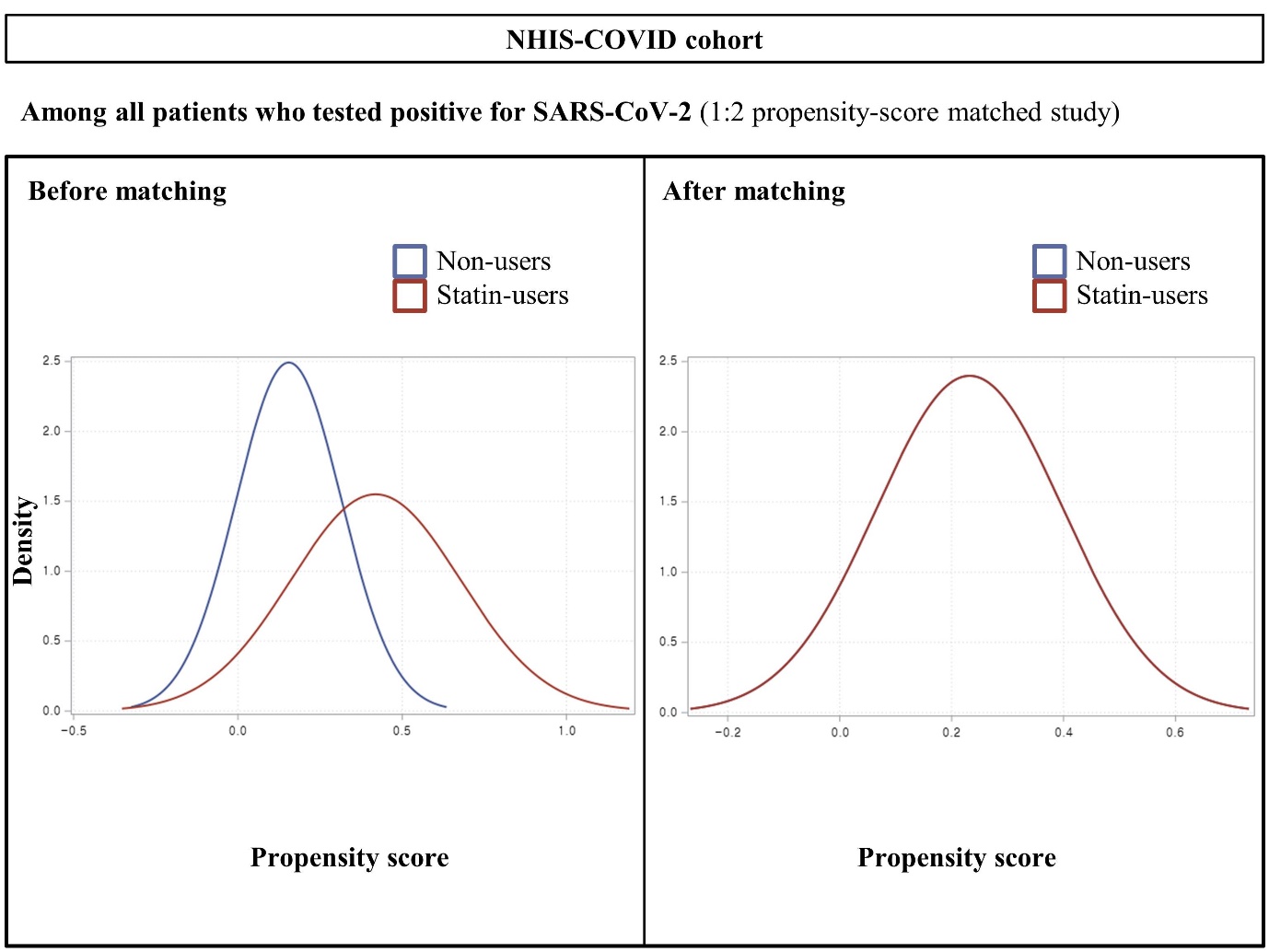


**Figure S5.** Disposition of patients in the GPCR-COVID cohort.


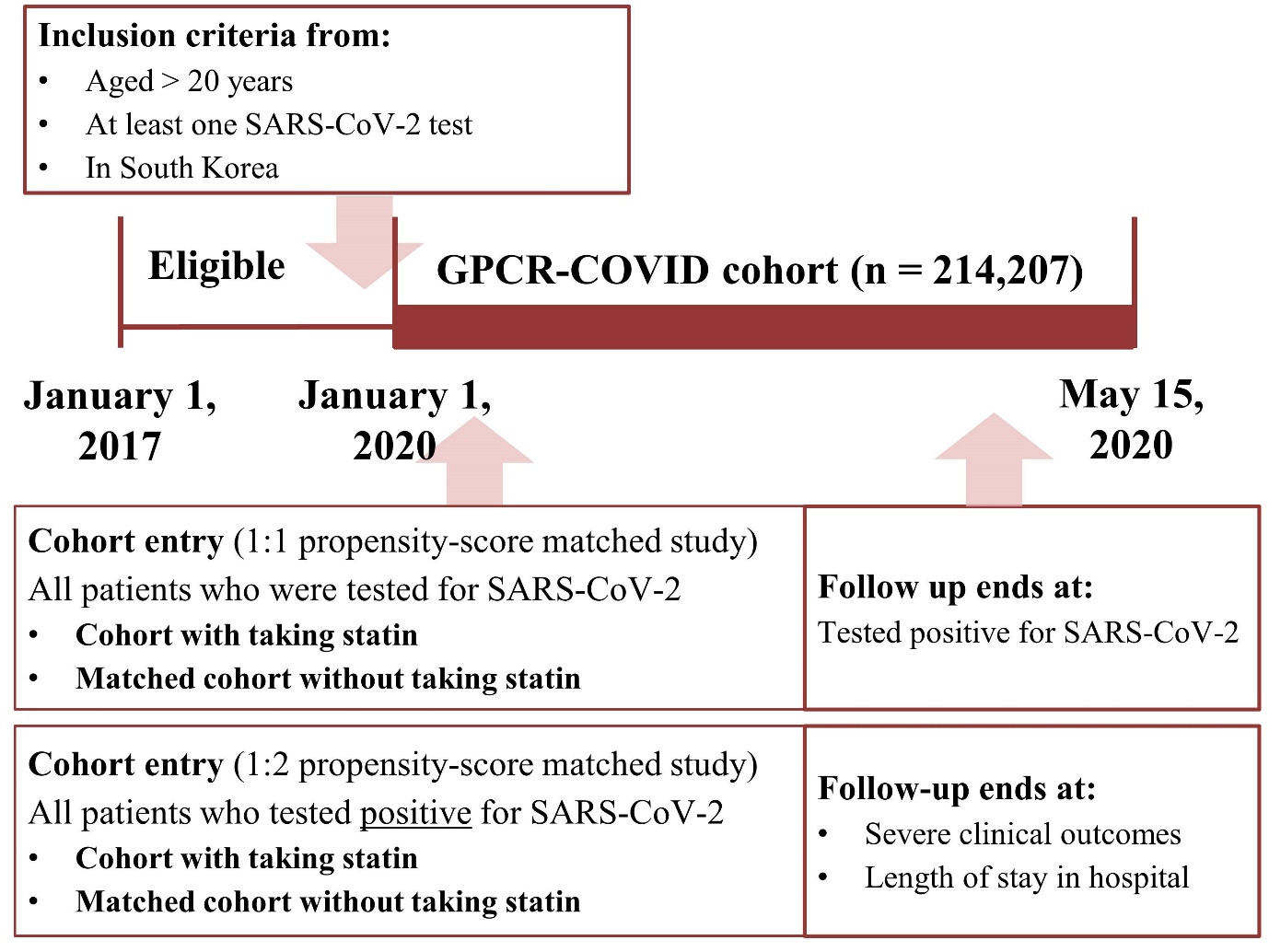


**Figure S6.** Disposition of patients in the NHIS -COVID cohort.


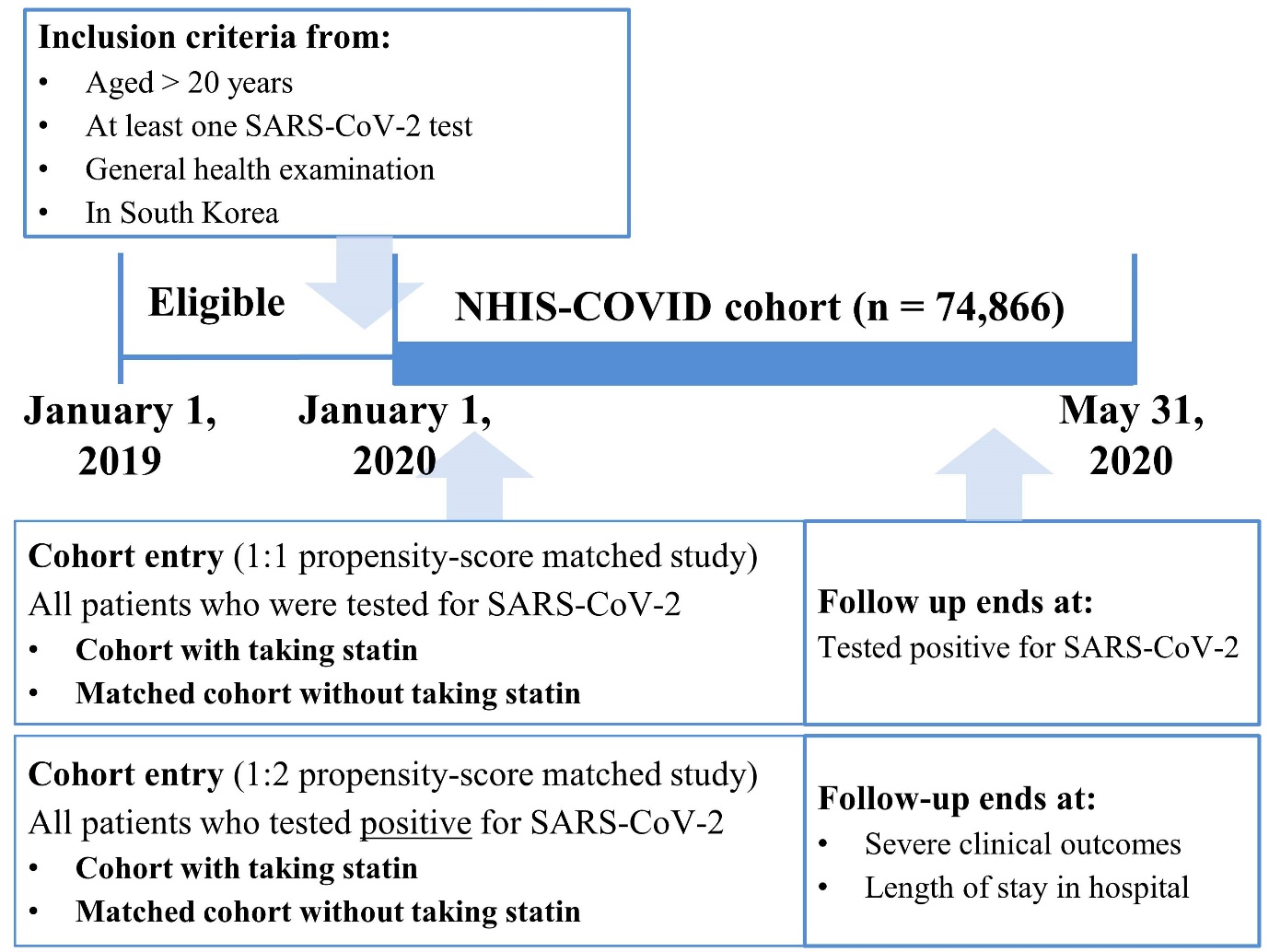

Supplement: Multimedia Appendix 1 [file publichealth_v7i10e29379_app1.docx]
